# Large cribriform growth pattern identifies ISUP grade 2 prostate cancer at high risk for recurrence and metastasis

**DOI:** 10.1038/s41379-018-0157-9

**Published:** 2018-10-22

**Authors:** Eva Hollemans, Esther I. Verhoef, Chris H. Bangma, John Rietbergen, Jozien Helleman, Monique J. Roobol, Geert J.L.H. van Leenders

**Affiliations:** 1 Department of Pathology, Erasmus MC Cancer Institute, University Medical Center, Rotterdam, The Netherlands; 2 Department of Urology, Erasmus MC Cancer Institute, University Medical Center, Rotterdam, The Netherlands; 3 Department of Urology, Franciscus Gasthuis & Vlietland, Rotterdam, The Netherlands

**Keywords:** Prostate cancer, Prostate cancer, Disease-free survival

## Abstract

Invasive cribriform and intraductal carcinoma are associated with adverse clinical outcome in patients with Gleason score 7 prostate cancer. It is yet unclear whether invasive cribriform and intraductal carcinoma of the prostate both have independent prognostic value, or whether field size of invasive cribriform carcinoma has impact on disease outcome. Our objective was to determine the prognostic impact of intraductal and invasive cribriform prostate cancer histological subtypes in radical prostatectomies. We reviewed 420 prostatectomy specimens with ISUP grade 2 prostate cancer, assessed the percentages of Gleason grade 4 and tertiary 5, and performed immunohistochemistry for basal cells to discriminate intraductal from invasive cribriform growth. Small and large invasive cribriform fields were distinguished based on a diameter of at least twice the size of adjacent pre-existent normal glands. Clinicopathological parameters and biochemical recurrence-free survival were used as endpoints. Cribriform architecture was observed in 228 (54.3%) men, 103 (24.5%) of whom had intraductal, 194 (46.2%) small invasive, and 34 (8.1%) large invasive cribriform growth. Large invasive cribriform architecture was associated with older age (*P* < 0.001), higher percentage Gleason grade 4 (*P* = 0.001), extraprostatic expansion (*P* < 0.001), and more frequent lymph node metastases (*P* = 0.002), when compared with small invasive cribriform and/or intraductal carcinoma. Univariate analysis identified PSA, pT-stage, surgical margin status, and intraductal and invasive cribriform growth as significant predictors for biochemical recurrence-free survival. In multivariable Cox regression analysis, pT-stage (hazard ratio = 1.64, 95% CI: 1.02–2.63, *P* = 0.04), positive surgical margins (hazard ratio = 3.28, 95% CI: 2.06–5.23, *P* < 0.001), and large cribriform growth (hazard ratio = 4.36, 95% CI: 2.08–9.17, *P* < 0.001) were independent predictors for biochemical recurrence-free survival, while intraductal carcinoma, small cribriform growth, and percentage of Gleason grade 4 were not. In conclusion, large cribriform fields represent an aggressive subpattern of invasive cribriform prostate cancer and are an independent predictive factor for biochemical recurrence-free survival in ISUP grade 2 prostate cancer patients.

## Introduction

The Gleason score is one of the most important parameters for clinical decision-making in men with prostate cancer. The Gleason grading system is entirely based on tumor architectural growth patterns which are classified into five different grades. While men with biopsy Gleason score 6 are frequently eligible for active surveillance, treatment is warranted in patients with Gleason score 8–10. The optimal therapeutic strategy for individual patients with Gleason score 7 is not yet clear. While most patients with Gleason score 7 undergo radical prostatectomy or radiation therapy, active surveillance is increasingly being considered in this large group of men. Therefore, there is an urgent need for additional parameters to aid therapeutic decision-making in men with Gleason score 7 prostate cancer.

Gleason score 7 prostate cancer is composed of well-delineated Gleason grade 3 glands along with Gleason grade 4 structures. Gleason grade 4 prostate cancer is heterogeneous, comprising a range of growth patterns, categorized as poorly formed, fused, glomeruloid, and cribriform [1, 2]. These individual growth patterns are generally not specified in pathology reports, however, several studies have found that patients with invasive cribriform growth have a worse outcome than men without this pattern [3–9]. Among cribriform prostate cancers heterogeneity of architectural pattern is still present, with some areas being round and small, while others are large and confluent vastly exceeding pre-existent gland diameter [10, 11].

Intraductal carcinoma of the prostate is characterized by either cribriform or solid malignant epithelial proliferation, or loose cribriform and micropapillary formations of severely atypical cells, in pre-existent large acini and prostatic ducts, with preservation of basal cells [2]. Although intraductal carcinoma is formally not included in the Gleason score, numerous studies have linked intraductal carcinoma to more aggressive disease [11–16]. The presence of intraductal carcinoma ought thus to be routinely noted in pathology reports [2, 17].

Invasive cribriform Gleason grade 4 prostate cancer and intraductal carcinoma often coexist, and can be difficult to distinguish without the use of immunohistochemical staining of basal cells. At present, it is not clear whether invasive cribriform carcinoma and intraductal carcinoma both have independent prognostic value for prostate cancer, or whether invasive cribriform subpatterns have additional prognostic value [5, 18]. The objective of this study is to determine the outcome of invasive cribriform subpatterns and intraductal carcinoma in patients with ISUP grade 2 after radical prostatectomy.

## Materials and methods

### Patient selection

In total 854 patients were identified who had undergone radical prostatectomy for prostate adenocarcinoma at the Erasmus MC, Rotterdam, The Netherlands between 2000 and 2017. Men who had undergone hormonal, radiation or viral therapy (*n* = 19) prior to operation were excluded from this study [19]. After fixation in neutral-buffered formalin, the radical prostatectomy specimens were sectioned transversely and entirely embedded for diagnostic purposes. All slides and blocks were available for pathology review. The use of tissue samples for scientific purposes was approved by the institutional Medical Research Ethics Committee (MEC-2011-295 and MEC-2011-296). Samples were used in accordance with the “Code for Proper Secondary Use of Human Tissue in The Netherlands” as developed by the Dutch Federation of Medical Scientific Societies (FMWV, version 2002, update 2011).

### Pathologic evaluation

**Fig. 1:**
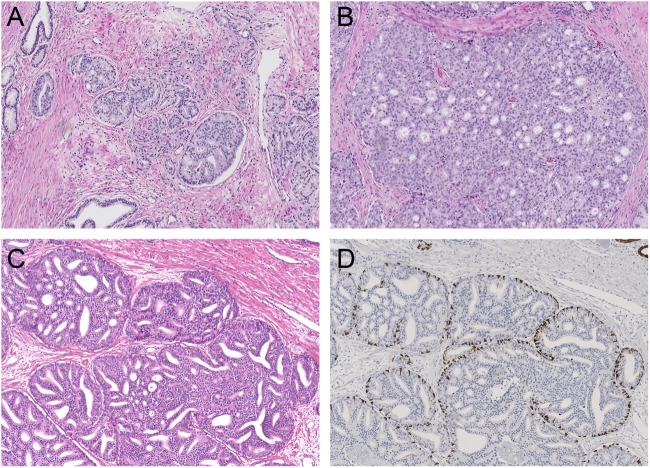
Gleason grade 4 cribriform growth patterns and intraductal carcinoma. **a** Small invasive cribriform carcinoma, 10×. **b** Large invasive cribriform carcinoma, 10×. **c**, **d** Intraductal cribriform carcinoma, with presence of basal cells, 10×

Two investigators blinded to clinical outcome (E.H. and Gv.L.) reviewed all radical prostatectomy specimens (*n* = 854). The following features were recorded: Gleason score according to the WHO/ISUP 2014 guidelines, pT-stage according to the AJCC TNM eighth edition, surgical margin status, presence of intraductal carcinoma, percentage Gleason grade 4, including specific growth patterns, and presence of tertiary Gleason grade 5 [2, 20]. Based on this revision, ISUP grade 2 specimen were identified. The following Gleason grade 4 growth patterns were recognized: poorly formed, fused, glomeruloid, and cribriform glands [2, 17]. In addition, we distinguished small and large cribriform growth patterns. Small cribriform structures had a diameter less than twice the size of adjacent benign glands. Large cribriform pattern was defined as having a diameter of at least twice the size of adjacent pre-existent normal glands, and could either represent one large well defined cribriform field or a confluent cribriform area (Fig. 1). Invasive cribriform Gleason grade 4 was morphologically distinguished from intraductal carcinoma based on the following features: invasive cribriform prostate cancer had irregular outline, showed anastomosing fields beyond pre-existent gland architecture or extension into periprostatic fat tissue, ejaculatory ducts, or seminal vesicles. Intraductal carcinoma was morphologically identified if cribriform structures were clearly continuous with pre-existent glands lined by normal basal epithelium, or containing corpora amylacea. Where invasive cribriform carcinoma and intraductal carcinoma could not be differentiated by morphological criteria alone, additional immunohistochemical staining for the presence of basal cells was performed; in total 261 slides from 156 ISUP grade 2 patients were stained. Gleason grade 5 was considered as a tertiary pattern if it occupied less than 5% of the total tumor area [1, 2, 17].

### Immunohistochemistry

Four micrometer thick tissue sections were cut from selected paraffin-embedded blocks and mounted on slides (Superfrost Microscopic Slides, ThermoFisher Scientific, Bleiswijk). Slides were deparaffinized and rehydrated with xylene and ethanol. Endogenous peroxidase was blocked using 0.3% H_2_O_2_ in phosphate-buffered saline. Heat-induced antigen retrieval was accomplished by 15 min in Tris-ethylenediaminetetraacetic acid buffer (pH 9; Klinipath, Duiven, The Netherlands). Mouse monoclonal high-molecular weight cytokeratin (clone 34BE12; 1:200; DAKO; Heverlee, Belgium) diluted in normal antibody diluent (APG-500; ScyTek Laboratories, West Logan, USA) was incubated for 2 h at room temperature. Antibody visualization was performed using the Envision kit (DAKO) and slide counterstaining with hematoxylin. When basal cell staining was completely absent around a cribriform gland, it was categorized as invasive cribriform carcinoma; if sporadic, scattered, or continuous basal cells were identified the structure was classified as intraductal carcinoma.

### Clinical follow up

Clinical follow-up after radical prostatectomy consisted of six-monthly, and later annual monitoring of serum prostate-specific antigen (PSA) levels. Biochemical recurrence was defined as PSA level ≥0.2 ng/ml measured at two separate time points at least three months apart when PSA had been undetectable after operation, or as PSA increase of >2.0 ng/ml if serum PSA had not declined to zero after operation. Postoperative lymph node and distant metastases were confirmed by biopsy or multidisciplinary consensus. Biochemical recurrence-free survival was defined as time in months from radical prostatectomy to biochemical recurrence.

### Statistical analysis

Normally distributed, continuous variables were analyzed using the independent sample Student’s *t* test, whereas variables without normal distribution were analyzed using the Mann–Whitney *U* test. Pearson’s chi squared (*χ*^2^) test was used for categorical parameters. Percentage Gleason grade 4 was analyzed both as continuous and dichotomous parameter (≥5% and <25% versus ≥25% and <50%). Missing PSA values (*n* = 27) were imputated using the median PSA value. Biochemical recurrence-free survival was analyzed using Cox proportional hazards regression and visualized by Kaplan–Meier curves, excluding patients with lymph node metastases at time of operation. Statistics were performed using SPSS version 24 (IBM, Chicago, IL, USA). Results were considered significant when the two-sided *P* value was <0.05.

## Results

### Patient characteristics

Out of the 854 revised patients, 420 showed ISUP grade 2 at radical prostatectomy and were included in this study. Median age at radical prostatectomy was 64.6 years (interquartile range: 59.8–68.1) and median PSA level was 8.2 ng/ml (interquartile range: 5.9–12.6). The tumor stage was distributed as follows: pT2 (*n* = 234; 55.7%), pT3a (*n* = 153; 36.4%), and pT3b (*n* = 33; 7.9%). A positive surgical margin was present in 142 cases (33.8%). In total 241 men (57.4%) had undergone pelvic lymph node dissection at the time of radical prostatectomy; in 12 patients (2.9%) one or more lymph node metastases were present.

Poorly formed glands (*n* = 325; 77.4%) were the most common Gleason grade 4 pattern followed by fused (*n* = 290; 69.0%), cribriform (*n* = 204; 48.6%), and glomeruloid (*n* = 194; 46.2%) glands. Seventy-five patients (17.9%) had one Gleason grade 4 pattern, 152 (36.2%) two, 133 (31.6%) three, and 60 (14.3%) four growth patterns. Tertiary grade 5 was present in 49 (11.6%) men.

In total, 228 (54.3%) patients showed either invasive or intraductal cribriform carcinoma. These patients had higher PSA levels (mean 12.2 ng/ml versus 9.4 ng/ml; *P* = 0.006) than those without cribriform architecture. They also more frequently had extraprostatic extension (51.8% versus 35.4%; *P* < 0.0001) and positive surgical margins (39.5% versus 27.1%; *P* = 0.007). One hundred and fifty (65.8%) patients with cribriform architecture and 91 (47.4%) patients without cribriform architecture had undergone pelvic lymph node dissection at the time of radical prostatectomy. Twelve of the (8.0%) patients with cribriform architecture were found to have lymph node metastasis at time of radical prostatectomy, compared to none in the group without cribriform architecture (*P* = 0.006).

### Comparison of invasive cribriform and intraductal carcinoma

Detailed histopathological and immunohistochemical analysis revealed that invasive cribriform carcinoma was present in 204 (48.6%), and intraductal carcinoma in 103 (24.5%) cases. Solid and loose papillary morphological variants of intraductal carcinoma were rarely observed, and co-existed with cribriform intraductal carcinoma in each case. Seventy-nine (18.8%) men had both intraductal carcinoma and invasive cribriform carcinoma, while 24 (5.7%) patients had intraductal carcinoma without invasive cribriform growth. Invasive cribriform growth without intraductal carcinoma was present in 125 men (29.8%). PSA levels (*P* = 0.06), pT stage (*P* = 0.32), surgical margin status (*P* = 0.36), and occurrence of lymph node metastasis (*P* = 0.39) were not statistically significant different between patients with invasive cribriform carcinoma without intraductal carcinoma (*n* = 125) and men with intraductal carcinoma only (*n* = 24). Patients with both invasive cribriform and intraductal carcinoma (*n* = 79) more frequently had extraprostatic extension (60.8% versus 46.4%; *P* = 0.02) and lymph node metastasis (11.4% versus 1.6%; *P* = 0.003) than those with invasive cribriform growth without intraductal carcinoma; there was no statistically significant difference in PSA level (*P* = 0.07), pT stage (*P* = 0.64), surgical margin status (*P* = 0.20), and lymph node metastasis (*P* = 0.32) between men with combined invasive cribriform and intraductal carcinoma and intraductal carcinoma only.

### Large invasive cribriform carcinoma

Large invasive cribriform growth was observed in 34 (8.1%) patients. All of these men (100%) had concomitant small invasive cribriform growth and 24 (70.6%) had intraductal carcinoma (Table 1). We compared patients with invasive large cribriform growth with men who had either small invasive cribriform growth and/or intraductal carcinoma (*n* = 194). The age of patients with large cribriform architecture (66.2 years; interquartile range: 63.3–70.9) was higher (*P* = 0.03) than of men with small cribriform architecture (63.8 years; interquartile range: 60.1–67.8). Albeit PSA levels of men with large cribriform architecture were higher (15.0 ng/ml; interquartile range: 8.3–18.3) than in those with small cribriform architecture (11.8 ng/ml; interquartile range: 6.0–13.4), this did not reach significance in this cohort (*P* = 0.16). In total 23/34 (67.7%) patients with large cribriform pattern had extraprostatic extension (pT3) as compared to 96/194 (49.0%) with small cribriform pattern (*P* < 0.001), although positive surgical margins were more frequently observed in the latter group (23.5% versus 42.3%; *P* = 0.04). The total percentage of Gleason grade 4 was 30.0% (interquartile range: 20–40%) in large and 23.3% (interquartile range: 15–30%) in small cribriform pattern (*P* = 0.001). Tertiary Gleason grade 5 was observed in 8/34 (23.5%) patients with large and 23/194 (11.9%) with small cribriform architecture, but this difference did not reach conventional measures of significance (*P* = 0.07). Lymph node metastases were observed in 6/26 (23.1%) men with large cribriform architecture and in 6/124 (4.8%) with small cribriform architecture (*P* = 0.002).

**Table 1:** Clinicopathological characteristics of ISUP grade 2 patients at radical prostatectomy

Characteristics	Noncribriform (*n* = 192)	Small cribriform (*n* = 194)	Large cribriform (*n* = 34)	*P* value
Age at time of RP (years)	63.2 (64.0; 59.2–68.1)	63.8 (64.6; 60.1–67.8)	66.2 (67.0; 63.3–70.9)	0.03^a^
PSA level (ng/ml)	9.4 (7.7; 5.4–10.5)	11.8 (8.3; 6.0–13.4)	15.0 (11.5; 8.3–18.3)	0.16^a^
*pT-stage (2009)*				
T2	124 (64.6)	99 (51.0)	11 (32.4)	<0.001^b^
T3a	63 (32.8)	78 (40.2)	12 (35.3)	
T3b	5 (2.6)	17 (8.8)	11 (32.4)	
T4	0	0	0	
Positive surgical margin	52 (27.1)	82 (42.3)	8 (23.5)	0.04^b^
Intraductal carcinoma	0	79 (40.7)	24 (70.6)	0.001^b^
*Intraductal versus invasive cribriform*				
IDC −/invasive cribriform−	192 (100.0)	0	0	<0.001^b^
IDC+/invasive cribriform−	0	24 (12.4)	0	
IDC−/invasive cribriform+	0	115 (59.3)	10 (29.4)	
IDC+/invasive cribriform+	0	55 (28.4)	24 (70.6)	
Tertiary Gleason 5	18 (9.4)	23 (11.9)	8 (23.5)	0.07^b^
PLND	91 (47.4)	124 (63.9)	26 (76.5)	0.16^b^
Lymph node metastasis	0	6 (4.8)	6 (23.1)	0.02^b^
Follow-up after RP (months)	62.6 (61.9; 14.6–100.2)	59.1 (41.3; 11.8–106.9)	53.2 (35.5; 13.2–73.4)	0.55^a^
BCR	29 (15.1)	48 (24.7)	18 (52.9)	0.001^b^
Distant metastasis	0	9 (4.6)	4 (11.8)	0.10^b^

Noncribriform cases do not have invasive cribriform carcinoma or intraductal carcinoma. Small cribriform cases include men with small invasive cribriform carcinoma and/or intraductal carcinoma. Large cribriform cases represent patients with presence of large invasive cribriform carcinoma, independent of the presence of small invasive cribriform carcinoma and intraductal carcinoma

Values denote either mean (median; interquartile range) or *n* (%). *P* values correspond to the comparison between small invasive cribriform and/or intraductal carcinoma and large invasive cribriform groups

^a^Student’s *t* test

^b^Pearson’s *χ*^2^ test

### Clinical outcome of invasive and intraductal carcinoma

**Fig. 2:**
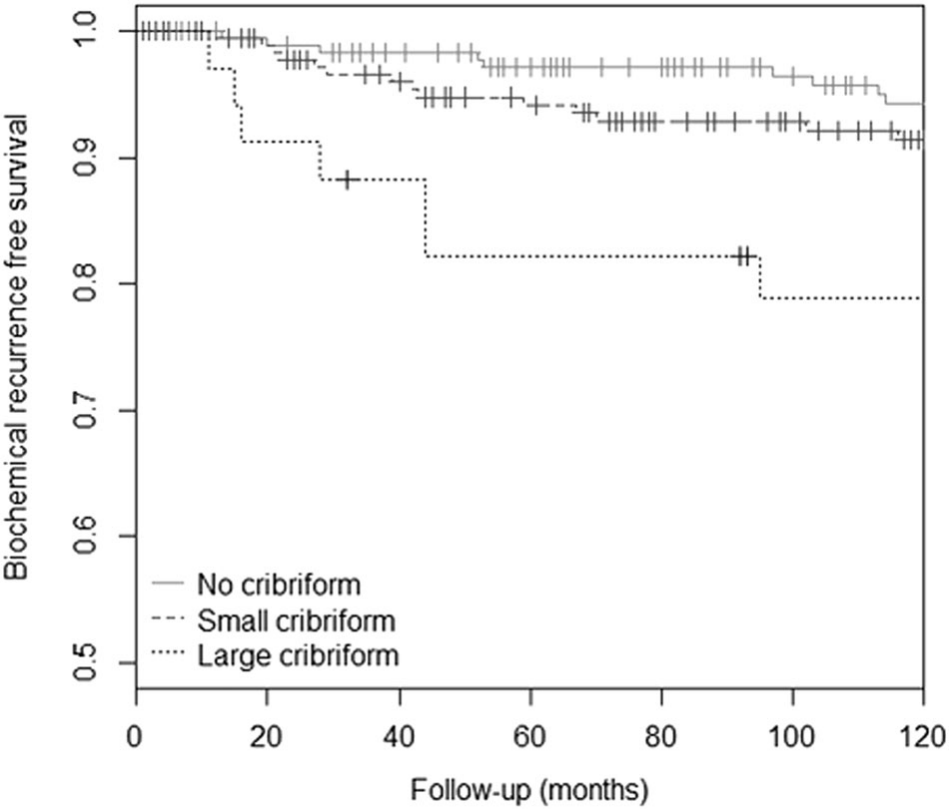
Biochemical recurrence-free survival of ISUP grade 2 patients, stratified for absent, small invasive and/or intraductal carcinoma, and large invasive cribriform architecture growth (*P* value < 0.001)

The median follow-up of ISUP grade 2 patients without positive lymph node dissection at time of radical prostatectomy (*n* = 408) was 53 months (interquartile range: 12.7–99.1). During follow-up 86 men experienced biochemical recurrence after a median of 26 (interquartile range: 10.7–47.6) months. Biochemical recurrence occurred more frequently (*χ*^2^, *P* = 0.01) in the large invasive cribriform (13/28; 46.4%) than in the small invasive cribriform and/or intraductal group (44/188; 23.4%), and was lowest in ISUP grade 2 patients without any cribriform growth (29/192; 15.1%, *P* = 0.04). The median time to biochemical recurrence was significantly shorter (log rank, *P* < 0.001) in patients with large invasive cribriform growth (11 months; interquartile range: 2.6–37.2) than in patients with small cribriform growth (25 months; interquartile range: 11.3–39.3) and no cribriform architecture (43 months, interquartile range: 15.4–73.8) (Fig. 2).

Univariate analysis showed that PSA (hazard ratio = 1.02, 95% CI: 1.01–1.04, *P* = 0.0001), pT3a (hazard ratio = 2.00, 95% CI: 1.27–3.14, *P* = 0.003), pT3b (hazard ratio = 4.42, 95% CI: 2.24–8.72, *P* < 0.001), positive surgical margins (hazard ratio 3.24, 95% CI: 2.11–4.97, *P* < 0.0001), intraductal carcinoma (hazard ratio = 2.13, 95% CI: 1.36–3.36, *P* = 0.001), and any invasive cribriform growth (hazard ratio 1.78, 1.16–2.74, *P* = 0.008) were all significant predictors for biochemical recurrence-free survival (Table 2). Percentage Gleason grade 4 was neither predictive as a continuous (hazard ratio = 1.01, 95% CI: 0.99–1.03, *P* = 0.076) nor as a dichotomized parameter (hazard ratio = 1.26, 95% CI: 0.82–1.93, *P* = 0.29). Tertiary Gleason grade 5 (hazard ratio = 1.29, 95% CI: 0.66–2.50, *P* = 0.46) did not have predictive value for biochemical recurrence in this cohort. In multivariable analysis, extraprostatic extension (pT3a, hazard ratio = 1.64, 95% CI: 1.02–2.63, *P* = 0.04), seminal vesicle invasion (pT3b, hazard ratio =  3.00, 95% CI: 1.42–6.34, *P* = 0.004), positive surgical margins (hazard ratio = 3.28, 95% CI: 2.06–5.23, *P* < 0.0001), and invasive large cribriform architecture (hazard ratio = 4.36, 95% CI: 2.08–9.17, *P* = 0.0001) were independent predictors for biochemical recurrence-free survival, while small invasive cribriform growth pattern and intraductal carcinoma were not. To determine whether the difference in prognostic value between invasive small and large cribriform growth could be explained by an overall higher percentage of cribriform growth, we compared the outcome of patients with ≥5% invasive cribriform growth and those with <5%. When invasive cribriform growth was present, no statistically difference existed between low- and high-cribriform percentage (log rank; *P* = 0.087).

**Table 2:** Cox regression analysis of biochemical recurrence-free survival in ISUP grade 2 prostate cancer patients without lymph node metastasis at time of operation (n = 408)

	Univariate analysis			Multivariable analysis		
	HR	95% CI	*P* value	HR	95% CI	*P* value
Age	0.99	0.96–1.03	0.56	0.99	0.95–1.03	0.57
PSA	1.02	1.01–1.04	<0.001	1.01	0.99–1.02	0.34
*pT-stage*						
T2						
T3a	2.00	1.27–3.14	0.003	1.64	1.02–2.63	0.04
T3b	4.42	2.24–8.72	<0.001	3.00	1.42–6.34	0.004
Positive surgical margin	3.24	2.11–4.97	<0.001	3.28	2.06–5.23	<0.001
Percentage Gleason 4	1.26	0.82–1.93	0.29	0.94	0.59–1.51	0.80
Tertiary Gleason 5	1.29	0.66–2.50	0.46	0.95	0.44–2.06	0.90
IDC-P	2.13	1.36–3.36	0.001	1.32	0.77–2.25	0.31
*Invasive cribriform*						
Small	1.50	0.95–2.37	0.09	1.07	0.65–1.75	0.80
Large	3.98	2.10–7.57	<0.001	4.36	2.08–9.17	<0.001

*HR* hazard ratio; *CI* confidence interval.

During follow-up 13 patients developed bone metastasis. Nine of these patients had small invasive cribriform or intraductal carcinoma (4.6%) and four had invasive large cribriform carcinoma (11.8%) at radical prostatectomy. The median time to bone metastasis was 138 months (interquartile range: 109.4–172.6) for small invasive and intraductal cribriform carcinoma and 59 months (interquartile range: 17.9–114.8) for invasive large cribriform carcinoma. Due to the low number of events we were not able to perform further statistical analysis.

## Discussion

While most patients with ISUP grade 2 prostate cancer are treated with radiotherapy and/or surgery, active surveillance is increasingly being considered as alternative strategy for these men [21–25]. Further risk stratification in this large group of patients is necessary to support therapeutic decision-making. Recently, invasive cribriform carcinoma and intraductal carcinoma have been recognized as promising additional predictive parameters for men with ISUP grade 2 prostate cancer [3–5, 11, 26]. In the current study, invasive and/or intraductal cribriform carcinoma was present in 54.3% of radical prostatectomies with ISUP grade 2 prostate cancer. While the clinicopathological features of men with invasive cribriform carcinoma without cribriform intraductal carcinoma were not statistically significant from those with cribriform intraductal carcinoma only, patients with both invasive and intraductal cribriform carcinoma more often had extraprostatic extension and lymph node metastasis than those with invasive cribriform carcinoma only. Furthermore, we found that patients with large invasive cribriform growth had higher pT-stage and more frequent positive lymph nodes than those with small invasive and/or intraductal cribriform carcinoma. In multivariable analysis, large invasive cribriform carcinoma was an independent predictor for biochemical recurrence-free survival, while small invasive carcinoma and intraductal cribriform carcinoma were not.

Various studies have addressed the association of either invasive cribriform carcinoma or intraductal carcinoma with adverse features at prostatectomy and with clinical outcome [3, 5, 8, 11, 27]. We observed that invasive and intraductal cribriform carcinoma were present in 48.6% and 24.5%, respectively of prostatectomy specimens with ISUP grade 2 prostate cancer. These rates are comparable to those found by others. Trudel et al. for instance found intraductal carcinoma in 17.5%, invasive cribriform carcinoma in 45.6%, and both invasive and intraductal cribriform carcinoma in 36.8% of 57 prostate specimen [11]. In a cohort of 286 ISUP grade 2 prostate cancer patients, Choy et al. demonstrated intraductal carcinoma in 26.5% and invasive cribriform growth in 38.7% [27]. Two studies took into account large cribriform architecture, however, these used different thresholds [6, 11]. Iczkowski et al. defined large cribriform pattern as having more than 12 luminal spaces, while area size exceeding the size of an average benign gland was used by Trudel et al. Our threshold of large cribriform fields as at least twice the size of normal adjacent glands, exceeds that of previous studies. For instance, in the study of Iczkowski et al. no cases were present with small cribriform pattern only, while small invasive cribriform carcinoma was present in 40% of our cases. To elucidate the clinical and biologic relevance of invasive cribriform and intraductal carcinoma in prostate cancer, it is crucial that it is clear how both entities are defined.

In a previous case–control study of 161 men with Gleason score 7 at radical prostatectomy, we found invasive cribriform but not intraductal carcinoma to be a significant predictive marker for metastasis- and disease specific-free survival in multivariate analysis [5]. In a subsequent analysis of prostate biopsies with long-term follow-up, both invasive and intraductal carcinoma had predictive value for disease-specific death, and combining both lesions had the strongest prognostic value [4]. The prognostic value of invasive and intraductal carcinomas at biopsies does not always correspond with the prognostic value at radical prostatectomies. Sampling artifacts inherently associated with diagnostic biopsies are likely the cause of discrepancies between biopsies and radical prostatectomy specimens. This is for instance reflected by the frequency of cribriform growth in biopsies and resection specimens; while invasive and/or intraductal cribriform architecture was found in 17% of sextant biopsies with ISUP grade 2, it was present in 54.3% of radical prostatectomy specimens in the current study [4]. Since most biopsy schedules currently include between 8 and 16 biopsies, and biopsies are increasingly being targeted by magnetic resonance imaging, the frequency of invasive cribriform, and/or intraductal carcinoma is higher with fewer sampling artifacts [28, 29]. Since both small cribriform growth and intraductal carcinoma are often associated with large cribriform growth, these patterns should still be reported.

The outcome of this study may have important implications. First, we propose the inclusion of the presence of large invasive cribriform in pathology reports. Of 26 men with large cribriform architecture who had undergone pelvic lymph node dissection at time of radical prostatectomy, 23% (*n* = 6) had lymph node metastasis. Men with large cribriform architecture should therefore not be considered for surveillance but instead be offered active treatment with lymph node dissection. On the other hand, the absence of metastasis and low risk of biochemical recurrence in Gleason score 3 + 4 = 7 patients with no cribriform architecture might indicate that active surveillance can be considered in these men, and that pelvic lymph node dissection might be omitted when treatment is offered. However, it is important to note that the current results were obtained after studying radical prostatectomy specimens, while treatment decisions are made based on diagnostic biopsies. An urgent need exists to incorporate pathological features such as small and large invasive cribriform growth, as well as intraductal carcinoma, into clinical nomograms and prediction tools.

Strong points of this study are the detailed histological review and the extensive immunohistochemical staining for classification of cribriform architecture. Although large cribriform growth is an adverse predictive parameter for ISUP grade 2 prostate cancer patients, the stringent cut-off used in this study resulted in the inclusion of a relatively small number of cases and must be validated. Finally, the retrospective study design and relatively short median follow-up of 53 months possibly gave rise to a selection bias.

In conclusion, we demonstrate that patients with large invasive cribriform growth represent a more aggressive subgroup of cribriform ISUP grade 2 prostate cancer. Men with large invasive cribriform carcinoma should be actively treated since they are at increased risk for biochemical recurrence and metastasis.

## Electronic supplementary material

Open License to Publish Form

Open Access Payment Form
