# Supplementary material for: Large cribriform growth pattern identifies ISUP grade 2 prostate cancer at high risk for recurrence and metastasis
Source: Mod Pathol. 2018 Oct 22;32(1):139–46. doi: 10.1038/s41379-018-0157-9 (PMC6300553; doi:10.1038/s41379-018-0157-9)
Supplement: Supplementary file 2 — Open Access Payment Form [file 41379_2018_157_MOESM2_ESM.pdf]

Modern Pathology

SPRINGER NATURE

OPEN ACCESS Article Processing Charge (APC) Payment Form

If you wish to take the open access option for your article, you must complete this payment form in addition to the Open Access Licence to Publish form. This will allow your manuscript (as both full-text HTML and PDF) to be made open access immediately upon publication, permanently available free of charge to all readers.

Completion of this form constitutes consent for the indicated article to be published on an open access basis in accordance with the Open Access Licence to Publish form and confirms you agree to the charges detailed below. Credit terms are 30 days from date of invoice. Failure to pay your invoice within the stated credit term will result in the open access status of the article being rescinded, with the article being published and placed behind the paywall. You may also be subject to restrictions on your ability to publish with Springer Nature or this title in the future, involvement of a third party debt collection agency and legal proceedings.

It is mandatory to send this completed form and the Open Access Licence to Publish form to the return address (details below) to ensure your paper is published open access. If you do not wish your article to be made open access it is not necessary to complete this form.

The APC will be charged at the following rate:

Licence Type	APC
**CC BY*:** CreativeCommons-Attribution	£2,500 / $3,200 / €2,900

VAT or local taxes will be added where applicable: for details please see www.nature.com/TaxInformation
Where authors publish open access in this journal through payment of an APC, any additional publication fees such as page or colour charges are waived.

* The Creative Commons Attribution (CC BY) Licence is preferred by many research funding bodies. We support use of this licence as it is recommended for maximum disseminaton and use of open access materials.

Other Creative Commons licences are available - Please contact the editorial office if you would like to discuss an alternative Creative Commons licence, quoting your manuscript number.

Manuscript Details (COMPLETE IN BLOCK CAPITALS)

Title of article:
Large cribriform growth pattern identifies ISUP grade 2 prostate cancer patients at high risk for recurrence and metastasis

Manuscript number:
MP-2018-0571

Corresponding author name:
Eva Hollemans

Corresponding author email address:
e.hollemans@erasmusmc.nl

Billing Details (COMPLETE IN BLOCK CAPITALS)

Contact name:
G.J.L.H van Leenders

Billing address:
Erasmus Mc Cancer Institute
Dept. of Pathology
P.O. Box 2040
3000 CA Rotterdam, The Netherlands

Postcode/ZIP:
3000 CA Rotterdam

VAT / GST/Tax Number:

Organisation name:
Erasmus MC

Email Address:
g.vanleenders@erasmusmc.nl

Tel Number:
+31 (0)10 704 43900, +31 (0)6 30004372

Payment Method:

☑ **Credit Card** *We will contact you via telephone to obtain your credit card details.*
Tel No: (if different form above)

☐ **Invoice** *An invoice will be sent by post and email. Payment is required within 30 days.*
PO Number (if required)

PLEASE RETURN YOUR COMPLETED PAYMENT FORM AND OPEN ACCESS LICENCE TO PUBLISH TO:
Dr Catherine Kethcham, Managing Editor **Email:** modpath@comcast.net
